# Expression of G1- epitope of bovine ephemeral fever virus in *E. coli* : A novel candidate to develop ELISA kit

**Published:** 2017-06-15

**Authors:** Fereshteh Yazdani, Mehran bakhshesh, Majid Esmaelizad, Zohre Azita Sadigh

**Affiliations:** 1 Department of Animal Virology, Research and Diagnosis, Razi Vaccine and Serum Research Institute, Agricultural Research, Education and Organization Extension (AREEO), Karaj, Iran; 2 Department of Biotechnology, Razi Vaccine and Serum Research Institute, Agricultural Research, Education and Organization Extension (AREEO), Karaj, Iran; 3 Department of Human Vaccine Production, Razi Vaccine and Serum Research Institute, Agricultural Research, Education and Organization Extension (AREEO), Karaj, Iran.

**Keywords:** BEFV, Escherichia coli, ELISA, G1- Epitope

## Abstract

Bovine ephemeral fever is an acute and arthropod-borne viral disease of cattle and water buffalo which occurs seasonally in most of the world tropical and subtropical regions. The epizootic feature of the disease has been reported in Iran with serious economic consequences. The surface glycoprotein G of bovine ephemeral fever virus (BEFV) is composed of 4 antigenic sites (G1-G4) and plays the main role for eliciting neutralizing antibodies and protective immunity. The G1 – epitope is a linear antigenic site and conserved among BEFV strains. In order to develop an ELISA test based on G1-epitope as coating antigen, this study was carried out to express the recombinant G1-epitope of BEFV in prokaryotic system. Using PCR and specific primers, a length of 88 amino acid of the G glycoprotein of BEFV including G1- epitope was amplified and cloned into the expression vector pGEX-4T-1, with the GST moiety. The recombinant plasmid (pGEX-4T-1-G1) was then transformed into *Escherichia coli* BL_21_ and expression of fusion protein was induced by 0.10 mM IPTG. The maximum expression of the fusion protein was obtained at 16 hr post induction as verified by SDS-PAGE electrophoresis, and it was also confirmed that this protein bearing G1- epitope is sufficiently biologically active to bind to anti-BEFV serum in western blot experiment.

## Introduction

Bovine ephemeral fever virus (BEFV) is an arthropod-borne virus which causes a disabling febrile infection of cattle and water buffalo. The disease is common in tropical and subtropical regions of Africa, Asia, Australia and the Middle East and is of major economic importance.^1^ The BEF is also an enzootic and periodically epizootic disease in Iran with considerable economic impact.^2^ The viral agent is the type species of the genus* Ephemerovirus* within the family of *Rhabdoviridae*. The *Ephemerovirus* genus also consists of closely related viruses such as Adelaide River and Berrimah viruses.^3^

BEFV encodes five structural proteins including a nucleoprotein (N), a polymerase-associated protein (P), a matrix protein (M), a large RNA-dependent RNA poly-merase (L) and a surface glycoprotein (G).^4^ The type specific G, a class I transmembrane glycoprotein, responsible for cell attachment and entry, comprises 4 antigenic sites (G1-G4) capable of inducing protective immunity in cattle.^5^^,^^6^ The highly conserved G1 site (17aa) is a linear antigenic site mapped to amino acid 487-503 of the 623 amino acid G protein and comprises two minimal B cell epitopes which appeared to be specific for BEFV.^5^^,^^7^ Thus, expression of this region in different systems has been exploited for designing an ELISA test with capability to detect sera against BEFV but not closely related viruses within the genus.^8^^-^^10^ However, virus neutralization (VN) test still remains the gold standard method for detecting anti - BEFV antibodies.^11^^-^^17^

In an initial attempt to develop a specific and inexpensive ELISA test, this research was carried out exploiting the bacterial expression system for production of a specific recombinant protein including the G1-epitope of BEFV as a coating antigen.

## Materials and Methods

**RNA extraction and reverse transcription. **Blood samples were collected from febrile cattle affected by BEFV.^2^ Viral RNA was extracted from whole blood using RNX^TM^-plus Kit (CinnaGen, Karaj, Iran). Extracted RNA was solved in RNase free water and subjected to reverse transcription (RT). The cDNA synthesis was carried out by RevertAid^TM^ first strand cDNA synthesis kit (Fermentas, Waltham, USA), the extracted RNA (6 μL), Oligo (dT) primer (1 μL) and DEPC- treated water (5 μL) were heated at 65 ˚C for 5 min and cooled on ice, then 5X reaction buffer (4 μL), 1 μL RiboLock RNase inhibitor^TM^ (20 U per μL; Fermentas), 2 μL of 10 mM dNTP Mix, 1 μL of Revert Aid^TM^ M-MuLV reverse transcriptase (200 U per μL; Fermentas) were added and the reaction reached to the final volume of 20 μL with DEPC- treated water. The mixture was put in a thermocycler at 25 ˚C for 5 min, followed by 42 ˚C for 60 min and 72 ˚C for 5 min.

**PCR amplification and construction of recombinant plasmid. **PCR reaction was carried out on BEFV cDNA using forward (5’ ATCTCGAG*GGATCC*GGAATGATCTTYGTR GAACC3′, *Xho I* and *BamHI* sites are underlined and illustrated in italic, respectively) and reverse (5’GTCTCGA GAAACCAACCTAYAACAGCAG 3′, *Xho I* site underlined) corresponding to amino acid 443-530 (88 aa) of the glyco-protein G based on the prototype strain (BB7721) of BEFV. The amplification was made in a total volume of 50 μL of reaction mixture containing 4 μL of cDNA, 5 μL of 10X *Pfu* buffer with MgSo_4_, 2 μL dNTP Mix (2 mM each), 2 μL of each primer (10 pmol) and 0.6 μL of *Pfu* DNA polymerase (2.5 U μL^-1^; Fermentas) and dionized H_2_O to total volume of 50 μL. Thermal cycling program was: 94 ˚C for 2 min followed by 35 cycles of 94 ˚C for 50 sec, 60 ˚C for 1 min, 72 ˚C for 30 sec and 3 min final extension at 72 ˚C.

The purified PCR product (286 bp) and pGEX-4T-1 vector were double digested with *XhoI* and *BamHI *enzymes, and the PCR product was cloned into the vector using T4 DNA ligase enzyme (Fermentas). The recombinant plasmid was transformed into competent *E. coli* 101 by heat and shock method. Following amplification and alkaline extraction, the correct insertion of the fragment into the vector was confirmed by colony PCR, restriction enzyme digestion (*Pst1 and BamH1*) and sequencing in both directions (Bioneer, Daejeo, South Korea). Thus, the recombinant plasmid (pGEX-4T-1-G1) and the empty plasmid (pGEX-4T-1) were transformed into competent *E. coli* BL_21_ as expression system. To find the optimum condition, expression was induced with 0.10 mM IPTG and 0, 3, 5 and 16 hr post induction at 37 ˚C. 

**SDS-PAGE and immunoblot analysis. **The cell lysate from *E. coli* BL_21_ containing expressed proteins were collected. They were mixed with sample buffer and boiled for 7 min. The samples (50 µL) were run on 10 % SDS-polyacrylamide gel and stained with Coomassie - blue (AppliChem, Darmstadt, Germany). For immunoblot analysis, the gels were transferred to nitrocellulose membranes by liquid transfer system. The membranes were then incubated in blocking buffer containing 5% BSA for 2 hr at room temperature with shaking followed by three times washing in phosphate buffer saline containing Tween 20 (PBST; Sigma, St Louis, USA). Then, the membrane was incubated for 16 hr in PBST containing different dilutions (1:2000, 1:1000, 1:500, 1:200 ) of primary antibody which was rabbit polyclonal antiserum against BEFV prepared in Department of Animal Virology, Razi Vaccine and Serum Research Institute, Karaj, Iran using Iranian isolate,^2^ and it was found to have > 1: 256 titer based on viral neutralization (VN) test.

After three times washing with PBST, the membrane was again incubated in PBST containing 1:2000 dilution of Horseradish peroxidase-conjugated goat anti- rabbit IgG (Dako, Glostrup, Denmark) as secondary antibody for 2 hr. The membranes were washed three times with PBST and were incubated in the substrate [5 mL 4-chloro-1-naphtol (Bio Basic Inc., Ontario, Canada) dissolved in 5 mL Methanol plus 15 µL H_2_O_2_ (35%)] with shaking for 20 min for visualization of specific bands.

## Results

**Fig. 1:**
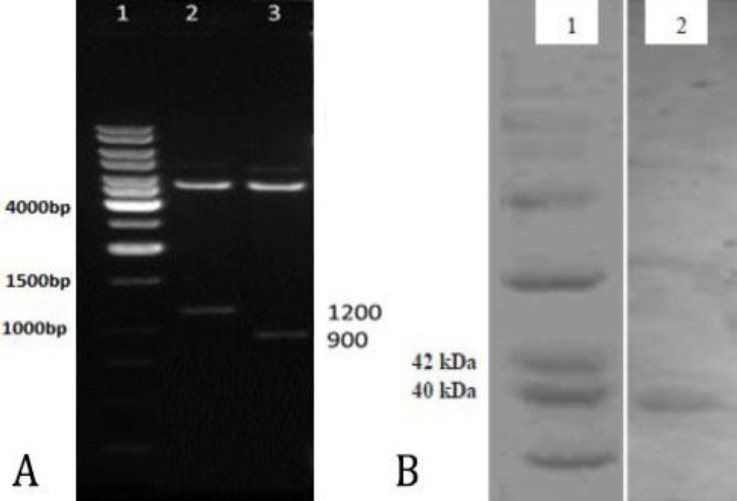
A) The recombinant (pGEX-4T-1-G1) and the empty plasmids (pGEX-4T-1) digested with restriction enzymes (*Pst1* and *BamH1*) producing 1200 bp and 900 bp fragments for the recombinant and empty plasmids, respectively. Lane 1: Ladder, Lane 2: Digested empty plasmid, Lane 3: Digested recombinant plasmid; B) Western blot analysis of the G1-GST fusion protein using rabbit anti-BEFV serum. 1) Mid-Range molecular weight protein marker; 2) The expected band (G1-GST fusion protein) appeared ~ 39 kDa

**PCR amplification and cloning. **The PCR reaction was carried out, the amplicon was run on the agarose gel and the correct band size (286 bp) was observed. The purified and restriction enzyme digested PCR products was cloned into pGEX-4T-1 plasmid to construct the recombinant pGEX-4T-1-G1 plasmid. The correct insertion of the fragment was confirmed by PCR colony while no band was seen for the empty plasmid. The recombinant plasmid (pGEX-4T-1-G1) and the control (pGEX-4T-1) were also confirmed by restriction enzyme digestion which resulted in 1200 bp and 900 bp fragments for the recombinant and control plasmids, respectively (Fig. 1A). 

**Fig. 2:**
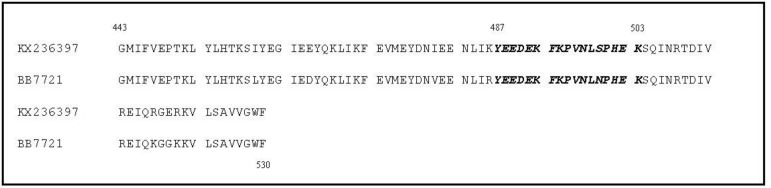
Protein sequence alignment (G^443^ to F^530^) of the Iranian BEFV isolate (KX236397) and the prototype strain (BB7721). The G1-epitope (Y^487^ to K^503^) illustrated in italic and bold

The accuracy of the cloned fragment was also approved by sequencing and the obtained sequence was deposited in the GenBank with accession number KX236397. Alignment of protein sequences of this isolate and the BB7721 (prototype) strain of BEFV including the G1- epitope (Y^487^ to K^503^) is illustrated in Figure 2. 

**Fig. 3:**
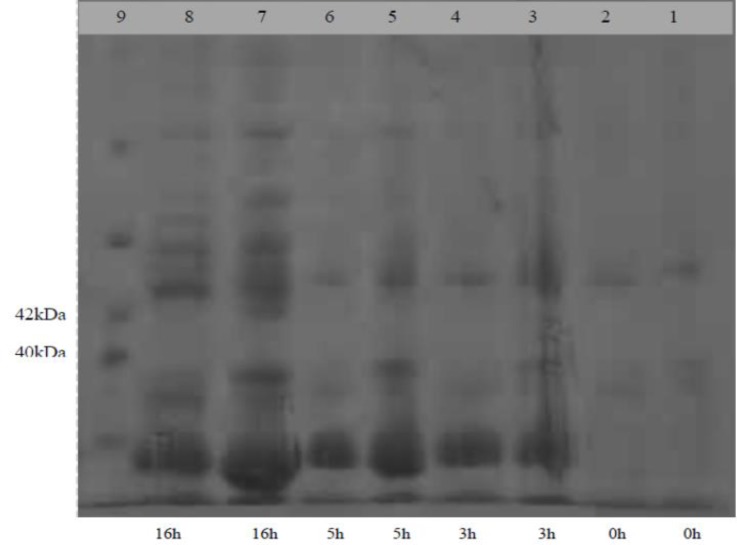
SDS-PAGE analysis of the expression products of the pGEX-4T-1 and pGEX-4T-1-G1 plasmids induced by 0.10 mM IPTG at 0, 3, 5 and 16 hr post induction. Lanes 1, 3, 5 and 7 are expression products of the recombinant plasmid (pGEX-4T-1-G1). Lanes 2, 4, 6 and 8 represent expression products of the empty plasmid (pGEX-4T-1

**Expression of G1 protein in** ***E. coli. ***Cell extracts from the recombinant and empty plasmids were analyzed at 0, 3, 5 and 16 hr post induction by SDS-PAGE, confirming that the G1-GST fusion protein was expressed in *E. coli*. Using Mid-range Marker (Promega, Madison, USA), low levels of the expected fusion protein (~39KDa) was detected in Coomassie-Blue-stained gel and the maximum expression condition was determined at 16 hr post induction (Fig. 3). Additionally, the correct expression and potential antigenicity of this novel recombinant protein (containing G1- epitope) to bind to BEFV antiserum (1:200 dilution) was verified by western blotting (BioRad, Hercules, USA) appearing an expected and unique band ~ 39 kDa as depicted in Fig. 1B. 

## Discussion

Bovine ephemeral fever has been known as a significant threat for cattle industry in Iran for the past decades, and due to the geographic situation of the country, epizootic feature of the disease occurs periodically with heavy economic consequences.^2^ Particularly for epidemiological studies and monitoring the immune status of vaccinated herds, a feasible and inexpensive test for detection of seropositive animal is an essential need. Viral neutralization test is currently the practical test, which has some limitations as well as low specificity to differentiate antibodies against BEFV from closely related viruses within the *Ephemerovirus *genus.^8^^,^^18^

A couple of research has been carried out in Australia and China to establish a practical ELISA test for detection of anti-BEFV specific antibodies.^8^^,^^9^ A blocking ELISA was developed using the whole G protein extracted from cell culture and anti-G1 antibody as coating antigen and monoclonal antibody, respectively. This ELISA was reported to be significantly specific to detect antibodies against BEFV but not Berrimah and Kimberely viruses.^8^ An indirect ELISA was also introduced using a glycosylated 140 amino acid of the virus glycoprotein including the G1-epitope expressed in *Pichia Pastoris *as coating antigen.^9^^,^^19^ Additionally, these authors developed an indirect ELISA exploiting the same amino acid fragment expressed in *E. coli* as the coating antigen.^10^^,^^20^ Evaluating a group of sera collected from seropositive and seronegative cattle, the both expression system illustrated high sensitivity and specificity compared to the viral neutralization test.

These studies also provided evidence that the both glycosylated and deglycosylated G1-epitopes were able to bind to anti-BEFV serum^19^^,^^20^ and consistent with the previous data^5^^,^^7^ again confirmed that the G1-epitope was a linear antigenic site. It has also been found that fusion recombinant G1 plus GST expressed in *E. coli* does not decline specificity of the indirect ELISA.^20^

Given these data, we selected a shorter length (88 amino acids) of the glycoprotein G the circulating BEFV in Iran including the G1-epitope. This novel recombinant protein which is expected to have less chance of cross reaction (increasing specificity) was successfully expressed in *E. coli* BL 21 as analyzed by SDS-PAGE. Using western blotting, it was also confirmed that this novel recombinant protein was biologically active to bind to anti-BEFV serum. Purification and application of this bacterially recombinant protein as the coating antigen will hopefully provide promising tool for development of an ELISA test with high sensitivity and specificity which is also benefited from advantage of large-scale and economic production of the coating antigen.

Additionally, potential immunogenicity of this easily producing protein to induce sufficient protective immunity against BEFV could be the subject of research designing recombinant vaccines in future.
